# Multiomic analysis identifies T cell subsets and mechanisms of epithelial interaction in idiopathic pulmonary fibrosis

**DOI:** 10.1172/jci.insight.203080

**Published:** 2026-09-08

**Authors:** Ana P.M. Serezani, Julia M.R. Bazzano, Bruno D. Pascoalino, Ludmilla da Silva, Abigail J. Dietrich, Chase J. Taylor, Taylor Sherrill, Annika Vannan, Carla L. Calvi, Paula I. Gonzalez-Ericsson, Erin M. Wilfong, Matthew Bacchetta, Ciara M. Shaver, Lorraine B. Ware, Margaret L. Salisbury, Luc Van Kaer, Nicholas E. Banovich, Jonathan A. Kropski, Timothy S. Blackwell

**Affiliations:** 1 Department of Medicine, Division of Allergy, Pulmonary and Critical Care Medicine, Vanderbilt University Medical Center, Nashville, Tennessee, USA.; 2 Translational Genomics Research Institute, Phoenix, Arizona, USA.; 3 Department of Medicine, Division of Hematology and Oncology,; 4 Department of Medicine, Division of Rheumatology and Immunology,; 5 Department of Cardiac Surgery, and; 6 Department of Pathology, Microbiology, and Immunology, Vanderbilt University Medical Center, Nashville, Tennessee, USA.; 7 Department of Cell and Developmental Biology, Vanderbilt University, Nashville, Tennessee, USA.; 8 Department of Veterans Affairs Medical Center, Nashville, Tennessee, USA.; 9 Department of Medicine, University of Michigan, Ann Arbor, Michigan, USA.; 10 Department of Veterans Affairs Medical Center, Ann Arbor, Michigan, USA.

**Keywords:** Immunology, Pulmonology, Adaptive immunity, Fibrosis

## Abstract

Idiopathic pulmonary fibrosis (IPF) is a fatal interstitial lung disease characterized by progressive scarring and respiratory failure. While T cells are elevated in IPF lungs, their contributions to fibrosis beyond inflammation remain poorly understood. Here, we performed multiplex imaging and single-cell RNA and protein profiling on about 90,000 CD3^+^ T cells from control and fibrotic lungs, revealing 11 distinct subsets of CD4^+^ and CD8^+^ T cells, including a rare CD56^+^ regulatory T cell. In addition to increased T cell numbers in severely fibrotic lungs compared with non-diseased controls, we observed CD4^+^ and CD8^+^ T cells localized near epithelial cells and in niches of abnormal epithelium. CXCR4/MIF signaling emerged as a central axis mediating T cell–epithelial interactions, while epidermal growth factor receptor (EGFR) and TGF-β pathways dominated in multiple T cell subsets. Our findings support the concept that T cells in IPF adopt nonclassical activation patterns that are driven by epithelial interactions within the fibrotic microenvironment. These studies provide a foundation for exploring alternative therapeutic strategies in IPF lungs by modulating T cell behavior and communication networks.

## Abstract 2

This study reveals features of epithelial/T-cell communication in idiopathic pulmonary fibrosis, shedding light on the contribution of T-cells in lung fibrosis.

## Introduction

Despite growing evidence that T cells play important roles in a wide range of chronic fibrotic disorders, including systemic autoimmune rheumatic disease–associated interstitial lung disease (SARD-ILD) (1–3), their role and functions in idiopathic pulmonary fibrosis (IPF) remain unresolved. In patients with IPF, T cell numbers are elevated in bronchoalveolar lavage fluid and lung tissues, and both CD4^+^ and CD8^+^ T cell subsets are found in greater abundance in regions of advanced fibrosis compared with areas with less severe fibrosis (4–7). In a previous study, we found that resident memory T cells (Trms), which are commonly located within epithelial barriers of mucosal tissues (8–10), are significantly elevated in IPF lungs compared with controls (11). These findings suggest that interactions between T cells and epithelial cells may be one of the major ways T cells contribute to fibrosis.

The lungs typically harbor various subtypes of T cells that are essential for maintaining the integrity of epithelial barriers (12–14). During fibrosis, alveolar epithelial cells undergo repeated injury with incomplete repair, resulting in prolonged activation of fibroblasts with accumulation of collagen and other extracellular matrix proteins. This process, which is influenced by factors such as environmental exposures, aging, and genetic variations (15–17), also results in the emergence of aberrant epithelial cell populations (18, 19), increased apoptosis, and cellular senescence (20–23). The role of T cells within this environment, and specifically their influence on aberrant epithelial cells, remain poorly understood. Elucidating these interactions could offer critical insights into the underlying mechanisms of fibrosis. In this study, we sought to investigate T cell subsets that are differentially activated in fibrotic lungs and to define their interactions with epithelial cells in the lung parenchyma.

Emerging single-cell technologies provide promising opportunities to characterize transient cellular interactions to understand the pathogenesis of diseases (24, 25). We applied cellular indexing of transcriptomes and epitopes by sequencing (CITE-seq) to lung-isolated T cells to evaluate subsets with relevant activation in the lungs of IPF patients compared with controls, and in parallel, we compared the activation of these subsets in other forms of ILD. We then used multiplex immunofluorescence by CODEX (co-detection by indexing) and spatial transcriptomic data (26) to investigate the distribution and distance of T cells and epithelial cells in fibrotic lungs, and single-cell RNA sequencing to analyze ligand-receptor pairs that mediate interactions between epithelial cells and T cells. Together, these studies provide a deeper characterization of T cells in fibrosis and reveal how proximity and interactions with the epithelium are central to shaping T cell activation in IPF lungs.

## Results

### Characterization of lung T cell subsets in IPF.

**Figure 1:**
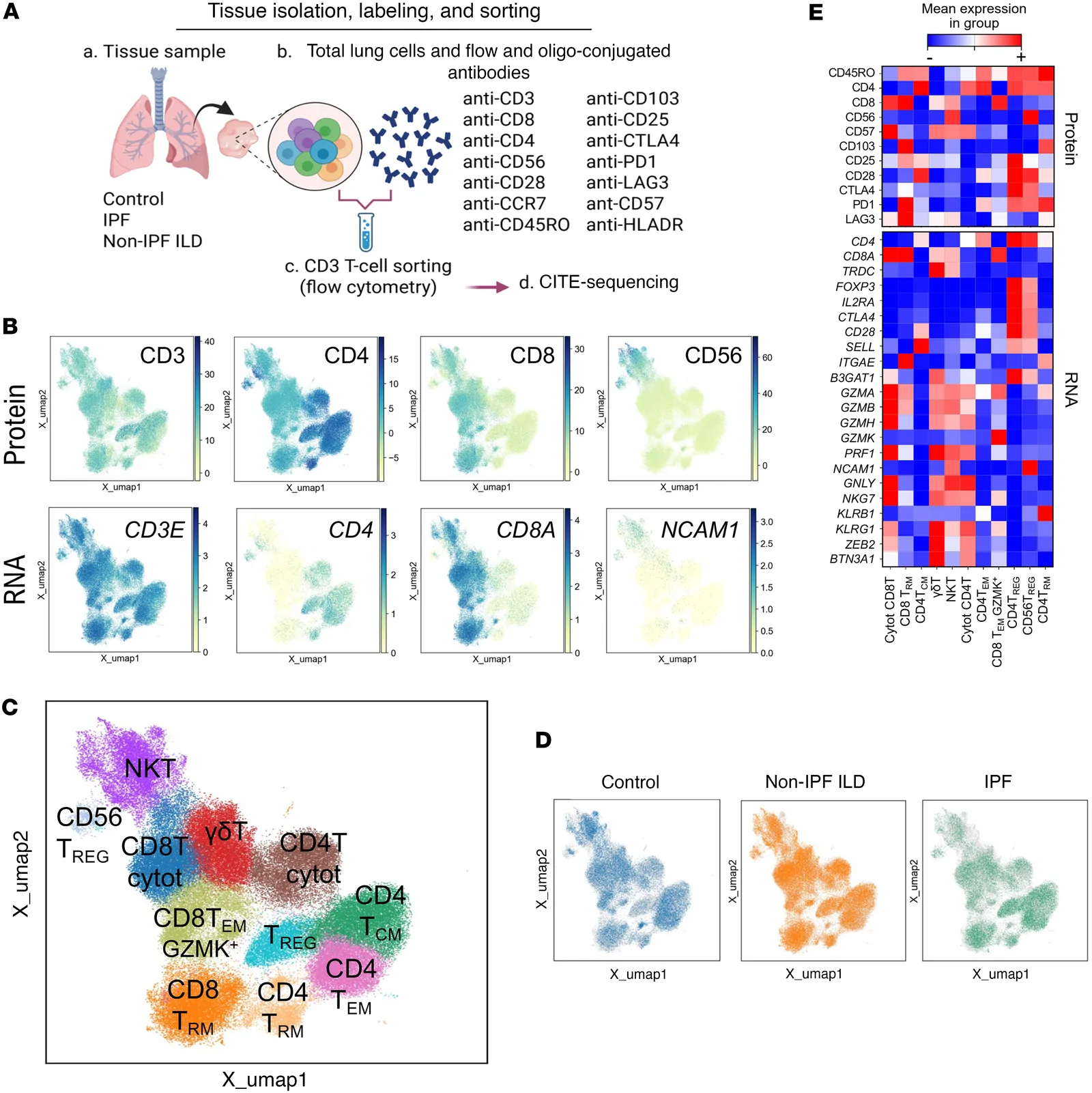
CITE-seq profiling and annotation of lung T cells. (**A**) Workflow schematic illustrating lung tissue dissociation, T cell staining, sorting, and CITE-seq library preparation. Created with BioRender (biorender.com). (**B**) Multimodal clustering integrating RNA and antibody-derived tag (ADT) protein measurements, visualized by uniform manifold approximation and projection (UMAP), from 91,434 cells isolated from 10 control lungs and 19 fibrotic ILD lungs (IPF and non-IPF ILD). Each dot represents one cell. (**C**) UMAP showing annotated T cell subsets based on combined RNA and ADT marker expression; colors indicate subsets as labeled. (**D**) UMAPs showing T cell cluster distribution stratified by diagnosis (control, non-IPF ILD, and IPF: *n* = 10, *n* = 10, and *n* = 9 lungs, respectively). (**E**) Heatmap of selected signature markers used to define each T cell subset, showing both RNA expression and ADT protein levels (values are normalized and scaled within each marker).

To identify the subsets of T cells present in the lungs, we performed cellular indexing of transcriptomes and epitopes by sequencing (CITE-seq) using the 10x Genomics platform on CD3^+^ T lymphocytes isolated from lung explants of 10 control patients, 9 IPF patients, and 10 non-IPF ILD patients (Supplemental Table 1; supplemental material available online with this article; https://doi.org/10.1172/jci.insight.203080DS1). Minced lung tissues were processed as previously described (11), and total CD3^+^ live T cells were sorted by flow cytometry. Before sequencing, cells were incubated with oligo-conjugated antibodies targeting 15 surface markers for CITE-seq (Figure 1A and Supplemental Table 2).

Using multimodal analysis of the processed data, we identified 11 subpopulations of CD4^+^ and CD8^+^ T cells based on combined RNA and protein marker expression (Figure 1B). Clusters were annotated according to canonical T cell markers as follows: CD4^+^ central memory T cells (Tcms; *SELL*^+^), CD4^+^ effector memory T cells (Tems; *CD45RO^+^SELL^–^*), CD4^+^ regulatory T cells (Tregs; CD25^+^, *FOXP3^+^*), CD4^+^ resident memory T cells (Trms; CD103^+^, *ITGAE^+^*), an unusual population of CD56^+^ Tregs (CD56^+^, *NCAM1^+^*, *FOXP3^+^*), CD8^+^ Tems (*GZMK^+^*), CD8^+^ Trms (CD103^+^, *ITGAE^+^*), cytotoxic CD4^+^ and cytotoxic CD8^+^ T cells (*GNLY^+^*, CD56^–^, *NCAM1^–^*), γδ T cells (*TRDC^+^*), and natural killer–like T cells (NKT-like; *GNLY^+^*, CD56^+^, *NCAM1^+^*) (Figure 1, C–E). Several of these subsets expressed cytotoxicity-related genes, including granzymes (*GZMA*, *GZMB*, *GZMH*). All T cell subsets were identified across participants and within the 3 groups, though in varied frequencies (Supplemental Figure 1, A and B). To confirm the existence of CD56^+^ Tregs in the lungs, we used our cytometry by time of flight (CyTOF) dataset from both peripheral blood and lungs (11) and identified the presence of FOXP3/CD56-expressing CD4^+^ T cells. CD56 was weakly detected in the lungs, but CD38 (a marker to identify activated NK cells; ref. 27) colocalized with FOXP3 in a subset of immune cells. These results indicate the presence of a CD56^+^ Treg-like population among CD4^+^CD25^+^ T cells, present predominantly in the lungs of IPF compared with control donors or peripheral blood (Supplemental Figure 1C). These findings highlight a diverse network of T cell subsets in the lung, including rare populations of regulatory T cells.

We explored disease-related alterations in gene expression between CD4^+^ Tregs and CD56^+^ Tregs by performing cell type–specific, subject-level pseudobulk differential expression analyses. We first identified differentially expressed genes (DEGs) in CD4^+^ Tregs and CD56^+^ Tregs in IPF compared with control subsets (Supplemental Figure 2A). We observed that the two subsets contain 33 common DEGs in IPF. These shared genes include canonical Treg markers, such as *FOXP3*, *IL2RA* (CD25), tumor necrosis factor receptor superfamily member 18 (*TNFRSF18*; also known as glucocorticoid-induced TNFR-related protein [GITR]), and T cell immunoglobulin and ITIM domain (*TIGIT*) (Supplemental Figure 2B). We performed a gene ontology analysis (Reactome pathway database, ref. 28) of the shared or unique DEGs observed in CD4^+^ Tregs and CD56^+^ Tregs (Supplemental Figure 2C), which revealed that shared genes are primarily involved in the regulation of the immune system and cytokine signaling, but also involved in other functions, such as the metabolism of carbohydrates. In contrast, DEGs unique to CD56^+^ Tregs are associated with several distinct pathways, which align with a more complex transcriptional response (Supplemental Figure 2C). We inspected the top 7 unique genes in CD56^+^ Tregs and observed that several genes were downregulated in IPF compared with control cells (Supplemental Figure 2D), including *EPHA4* (ephrin type A receptor 4), which is primarily expressed by Tregs in the immunosuppressive tumor microenvironment (29). As a comparison, we performed gene ontology analysis in Tregs isolated from autoimmune-associated ILD (non-IPF ILD) and controls and observed some overlap between pathways differentially regulated in these cell types in IPF; however, CD4^+^ Tregs in non-IPF ILD exhibited more DEGs and pathways compared with CD4^+^ Tregs in IPF lungs (Supplemental Figure 2, E–H).

### Defining the activation profile of T cells in IPF and non-IPF ILD.

**Figure 2:**
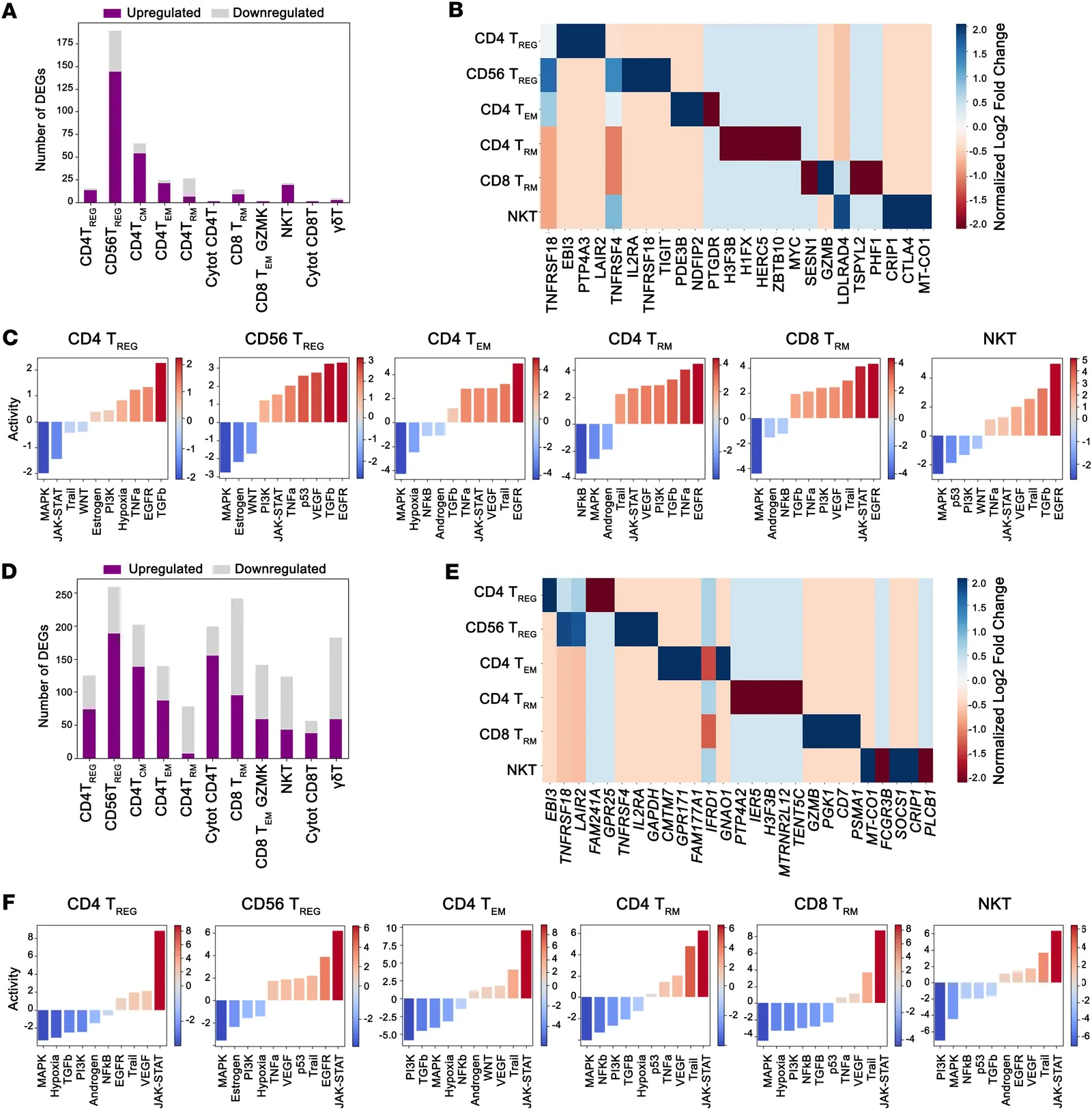
Differential gene expression and pathway activity across lung T cell populations in lung fibrosis. (**A**) Number of upregulated (purple) and downregulated (gray) differentially expressed genes (DEGs) in each T cell subset in IPF lungs compared with controls. DEGs were identified by PyDESeq2 pseudobulk analysis using thresholds of adjusted *P* value (padj) < 0.05 and log_2_ fold change > 1. (**B**) Heatmap of the top 5 DEGs per T cell subset from the IPF-versus-control pseudobulk analysis. (**C**) PROGENy pathway activity scores for the top 10 pathways across 6 T cell subsets in IPF lungs, inferred from pseudobulk gene expression profiles using multilevel modeling (MLM). Pathways are ranked by activity score; active pathways are shown in red and inactive pathways in blue. (**D**) Number of upregulated (purple) and downregulated (gray) DEGs in each T cell subset in non-IPF ILD lungs compared with controls (PyDESeq2 pseudobulk; padj < 0.05 and log_2_ fold change > 1). (**E**) Heatmap of the top 5 DEGs per T cell subset from the non-IPF ILD–versus–control pseudobulk analysis. (**F**) PROGENy pathway activity scores for the top 10 pathways across 6 T cell subsets in non-IPF ILD lungs, inferred by MLM from pseudobulk gene expression profiles; active pathways are shown in red and inactive pathways in blue.

To identify signaling pathways underlying different functions of T cells in fibrosis, we expanded our pseudobulk differential expression analyses to include all 11 T cell subsets. The large majority of DEGs were upregulated in T cells in IPF compared with control subsets (Figure 2A). CD56^+^ Tregs showed the greatest number of DEGs compared with other T cell subsets. Figure 2B shows the top 5 DEGs for CD4^+^ Tregs, CD56^+^ Tregs, CD4^+^ Tems, CD4^+^ Trms, CD8^+^ Trms, and NKT-like cells. In these T cell subsets, we further examined DEGs using a multivariate linear model and PROGENy (a pathway inference framework based on different pathway resources; ref. 30) to identify differentially activated pathways. We found that epidermal growth factor receptor (EGFR) signaling, TGF-β, and TNF/TRAIL signaling were increased in these T cell subsets, with EGFR signaling consistently being the most upregulated pathway across subsets (Figure 2C). These results indicate a conserved set of signaling pathways in IPF, which are likely influenced by local factors present in the fibrotic microenvironment.

Next, we analyzed T cell DEGs in non-IPF ILD subjects compared with control samples. We observed that DEGs were identified in all subsets of T cells in non-IPF ILD compared with controls, with the majority of DEGs being upregulated (like IPF) (Figure 2D). In addition to the finding of more DEGs in non-IPF ILD compared with IPF, the top 5 DEGs in non-IPF ILD subjects overlapped with but were not identical to those in IPF patients (Figure 2E). Importantly, upregulated pathways in CD4^+^ Tregs, CD56^+^ Tregs, CD4^+^ Tems, CD4^+^ Trms, CD8^+^ Trms, and NKT-like cells were different from those in IPF. Janus kinase and signal transducer and activator of transcription (JAK-STAT), TRAIL, and VEGF pathways were upregulated in all these T cell subsets in non-IPF ILD compared with controls (Figure 2F). Together, these results indicate important differences in the microenvironment of non-IPF ILD compared with IPF, driving different sets of disease-specific pathways in T cells.

### Mapping lymphocyte spatial distribution in IPF lungs using tissue imaging.

**Figure 3:**
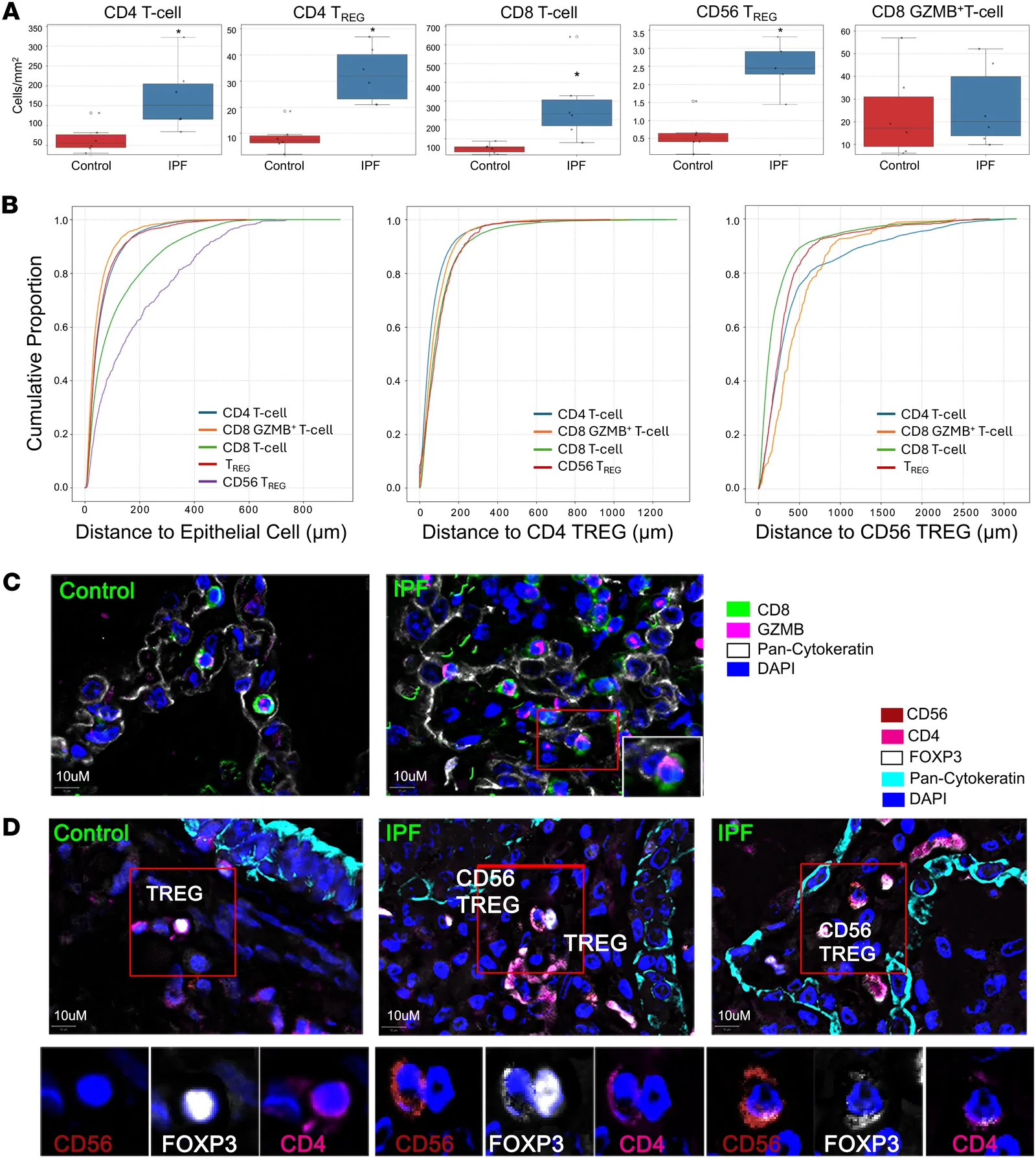
Spatial quantification of T cell subsets in control and IPF lungs by CODEX. (**A**) Cell types were annotated in QuPath as CD4^+^ T cells (CD4^+^), regulatory CD4^+^ T cells (Tregs; FOXP3^+^), CD8^+^ T cells (CD8^+^), cytotoxic CD8^+^ T cells (CD8^+^GZMB^+^), and CD56^+^ Tregs (CD56^+^FOXP3^+^). Box plots show normalized cell densities (cells/mm^2^) for each subset in control donors (*n* = 6) and IPF patients (*n* = 6); boxes indicate median and interquartile range, and points represent individual donors. Statistical significance was assessed using the Mann-Whitney *U* test (*P < 0.05). (**B**) Cumulative distribution of distances from each indicated T cell subset to the nearest epithelial cell in multiplexed spatial imaging of control and IPF lung tissues. (**C**) Representative CODEX images from lung tissue microarray sections showing CD8^+^GZMB^+^ T cells, stained for CD8 (green), granzyme B (GZMB; pink), pan-cytokeratin (white), and DAPI (blue). (**D**) Representative CODEX images from tissue microarray sections showing CD4^+^ Tregs and CD56^+^ Tregs, stained for CD56 (red), FOXP3 (white), CD4 (pink), pan-cytokeratin (aqua), and DAPI (dark blue). Scale bars: 10 μm.

To determine the relationship between T cells and epithelial cells in IPF, we examined the distribution of T cell subsets in the lungs using CODEX-multiplexed tissue. Tissue microarray sections were generated from 2 distinct regions of paraffin-embedded lungs from 6 non-diseased controls and 6 IPF patients (Supplemental Table 1). A panel of 14 canonical and functional markers (Supplemental Table 3) was used to identify immune cell subsets, epithelial cells (pan-cytokeratin positive), and endothelial cells (CD31^+^). Among T cells, we identified CD4^+^ T cells (CD4^+^), regulatory T cells (Tregs; forkhead box P3–positive [FOXP3^+^] and CD56^+^ Tregs [CD56^+^FOXP3^+^]), CD8^+^ T cells (CD8^+^), and CD8^+^ GZMB^+^ T cells (Figure 3A). Quantification of T cell subsets revealed a significant increase in CD4^+^ T cells, Tregs, CD8^+^ T cells, and CD56^+^ Tregs in IPF compared with controls. We also observed that T cells were localized primarily in the alveolar interstitium of both control and IPF lungs, with many T cells found in proximity to the epithelium (Supplemental Figure 3). Then, we measured the distance between these different types of T cells and the nearest epithelial cells (cytokeratin positive). A cumulative distribution plot revealed that CD4^+^ T cells, CD8^+^ GZMB^+^ T cells, and Tregs were the populations closest to epithelial cells, whereas other CD8^+^ T cells and CD56^+^ Tregs were positioned at greater distances from epithelial cells (Figure 3B). To further understand the differences between Tregs and CD56^+^ Tregs, we calculated distances between all T cell populations and these two subsets of regulatory cells. Tregs were localized near all T cell subsets, whereas CD56^+^ Tregs were closer to CD8^+^ T cells and CD4^+^ Tregs (Figure 3B), suggesting that CD56^+^ Tregs may have more limited cellular interactions compared with conventional Tregs. Figure 3, C and D, shows a representative image of CD8^+^ GZMB^+^ T cells and their proximity to epithelial cells, and proximity of Tregs and CD56^+^ Tregs, in lung tissue from control and IPF donors. While conventional Tregs expressed FOXP3 exclusively in the intranuclear compartment, we observed that CD56^+^ Tregs exhibited cytoplasmic localization of the transcription factor FOXP3 (Figure 3D). These findings indicate that certain T cell subsets, including CD4^+^ T cells, CD8^+^ GZMB^+^ T cells, and Tregs, are in proximity to epithelial cells and may play a role in influencing epithelial cell functions, behavior, and survival in fibrosis.

### Distribution of T cell subsets within distinct pathological niches.

To further evaluate T cells in regions of the lung with active epithelial remodeling, we utilized our Xenium spatial transcriptomics dataset (26). This dataset includes samples from non-fibrotic control donors and IPF donors separated into areas of mild or severe fibrosis. Pathological evaluation of sections that underwent transcriptomic analysis noted key disease-associated features, such as epithelial remodeling, hyperplasia, and epithelial detachment. We analyzed 10 unaffected samples and 35 samples from donors with pulmonary fibrosis diagnoses (including 16 IPF), quantified the mean nearest-neighbor distance from T cells to alveolar and airway epithelial cell types, and compared T cell abundance between unaffected control tissue and IPF regions with mild versus severe fibrosis.

**Figure 4:**
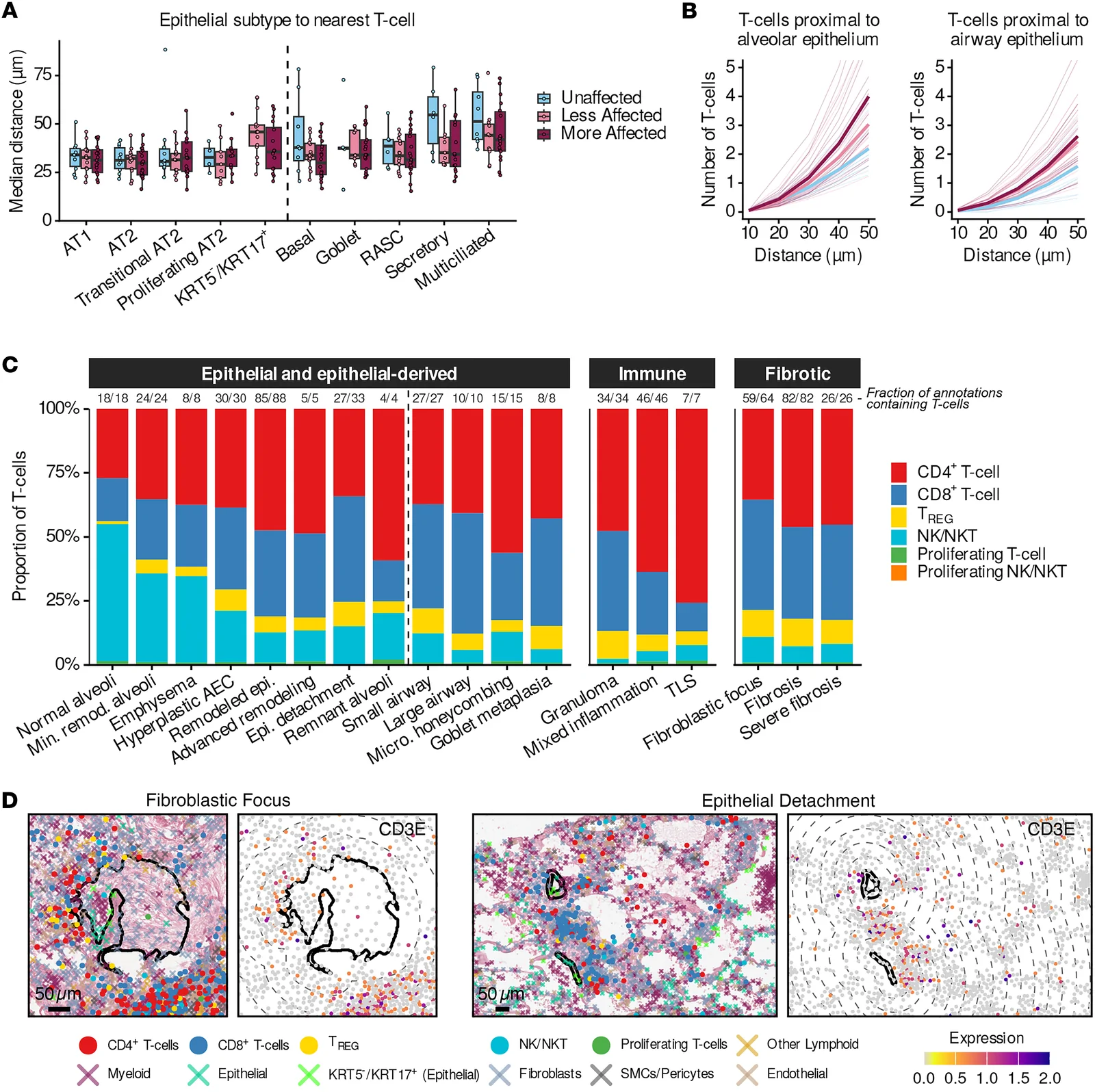
Spatial enrichment of T cell subsets in regions of epithelial abnormality and fibrosis. (**A**) Spatial transcriptomics analysis showing the median distance (micrometers) from each annotated epithelial cell subtype to the nearest T cell (CD3D/CD3E) in control (non-diseased) lung and in pulmonary fibrosis regions annotated as less affected or more affected. Each point represents one sample; box plots show the median and interquartile range across samples. (**B**) Cumulative mean number of CD4^+^, CD8^+^, Treg, NK/NKT, and proliferating T and NK/NKT cells located within 10–50 μm of alveolar or airway epithelial cells. Thin lines indicate individual samples and thick lines indicate group means. (**C**) Stacked bar plot showing the composition of T cells located inside and within 50 μm of representative normal and abnormal epithelial, immune, and fibrotic annotations. T cells within the range of multiple instances of the same annotation type were counted once. Fractions indicate the proportion of annotations of each type that contained T cells and were therefore included in the analysis. The dashed vertical line separates alveolar and airway epithelial annotation types. See Methods for full names of abbreviated annotation types. (**D**) Representative tissue annotations of fibroblastic foci and epithelial detachment showing the spatial distribution of T cell subsets within 50 μm of these structures. Adjacent panels show CD3E transcript expression in the same regions; solid lines denote the annotated regions and dashed rings indicate the approximately 50 μm perimeter. Scale bars: 50 μm.

These analyses showed that T cells (CD4^+^, CD8^+^, NK/NKT) are located in proximity to alveolar epithelial cells (alveolar type 1 [AT1], AT2, and transitional AT2 cells) in both control and affected lungs, and are also found near secretory, multiciliated, and basal epithelial cells in fibrotic lungs (Figure 4A). Using cumulative distribution analyses, we quantified T cells within 50 μm of epithelial cells and found that T cell numbers were higher in severely fibrotic regions compared with unaffected regions (Figure 4B), supporting a role for T cells in disease progression. In addition, we determined the proportion of CD4^+^ T cells, CD8^+^ T cells, Tregs, and NK/NKT cells in areas with specific pathological features and identified increased CD4^+^ and CD8^+^ T cells and a reduction in NK/NKT cells in most diseased regions compared with normal alveoli (Figure 4C). Figure 4D shows a representative image of the distribution of different T cell subsets within regions assigned as fibroblastic foci and epithelial detachment in IPF, which are enriched for KRT5^–^KRT17^+^ (aberrant basaloid) cells (dashed lines represent a 50 μm distance from the central area). Thus, these results further support the concept that epithelial-lymphocyte interactions could contribute to fibrosis.

### Molecular drivers of cell-to-cell communications between T cells and epithelial cells.

**Figure 5:**
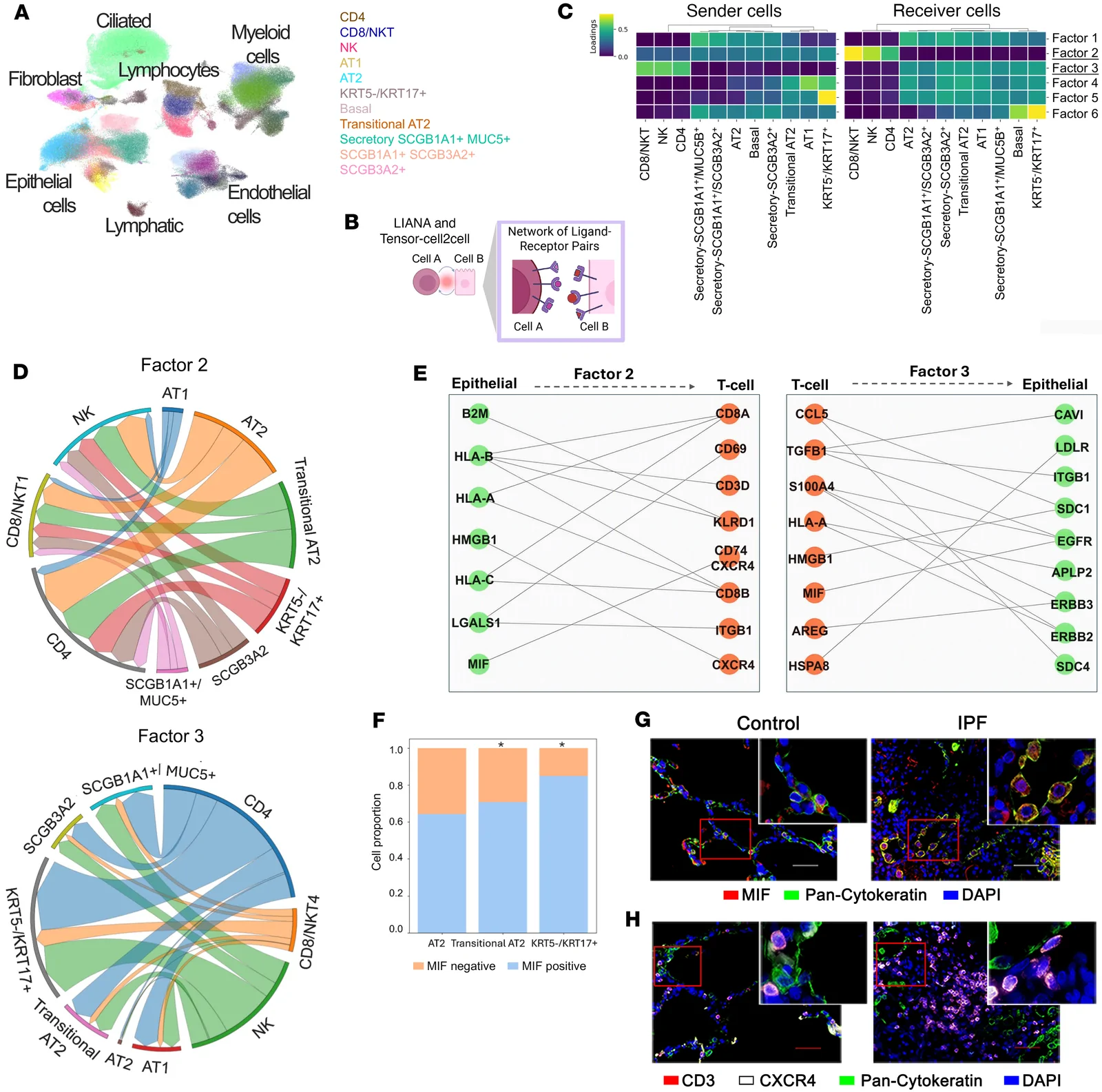
Cell-cell communication analysis of epithelial–T cell interactions in fibrotic lung. (**A**) UMAP of integrated whole-lung single-cell profiles from 224,404 cells isolated from 10 control lungs and 17 fibrotic ILD lungs. (**B**) Workflow schematic of ligand-receptor inference and tensor-based factorization to identify coordinated cell-cell interaction programs using LIANA and Tensor-cell2cell. Created with BioRender (biorender.com). (**C**) Heatmap summarizing sender and receiver cell populations participating in inferred interactions between the indicated T cell/NK subsets and epithelial populations based on LIANA and Tensor-cell2cell results. (**D**) Circos plot depicting epithelial–T cell relationships captured by interaction factors 2 and 3. (**E**) Bipartite network of significant ligand-receptor pairs associated with factor 2 (epithelial-to-T-cell signaling) and factor 3 (T-cell-to-epithelial signaling); nodes denote ligands and receptors, and edges represent interactions with weights greater than 0.005. (**F**) Stacked bar plot showing proportion of AT2, transitional AT2, and KRT5^–^KRT17^+^ cells classified as MIF positive (≥1 detected *MIF* transcript) or MIF negative (no detected *MIF* transcript) in all groups. Statistical significance was assessed using the Friedman test on per-library MIF-positive proportions, followed by post hoc paired Wilcoxon’s signed-rank tests (*P < 0.05). (**G** and **H**) Representative immunofluorescence images from lung tissue microarray sections (6 controls and 6 IPF): (**G**) MIF (red), pan-cytokeratin (green), and DAPI (dark blue); (**H**) CD3 (red), CXCR4 (white), pan-cytokeratin (green), and DAPI (dark blue). Scale bars: 100 μm.

To investigate how T cell/epithelial cell crosstalk may influence pulmonary fibrosis, we analyzed single-cell RNA sequencing (scRNA-seq) data from CD45^–^ (non-hematopoietic) and CD45^+^ (hematopoietic) lung cells generated from the same subjects who had undergone T cell CITE-seq analysis (23). The dataset was integrated using Harmony, resulting in 36 unsupervised clusters that we annotated as immune and structural cells, including lymphocytes; lymphatic, myeloid, epithelial, and basal cells; fibroblasts; and endothelial cells (Figure 5A). The lymphocyte cluster included CD4^+^, CD8^+^/NKT-like, and NK cells, and the epithelial cluster was composed of AT1, AT2, transitional AT2, and KRT5^–^KRT17^+^ cells and 3 subsets of secretory cells: SCGB1A1^+^MUC5^+^, SCGB1A1^+^SCGB3A2^+^, and SCGB3A2^+^. To identify the network of ligand-receptor pairs and the most common cell interactions between the different populations of T cells and epithelial cells, we used LIANA (ligand-receptor analysis framework) (31), a computational tool designed to identify interactions between secreted or cell membrane ligands and plasma membrane receptors across 2 different cells using multiomics data (Figure 5B). Additionally, we used Tensor-cell2cell analysis (32), an unsupervised method that generates matrices with communication scores for each ligand-receptor pair. This approach organizes the data into a 4-dimensional tensor structure, allowing for the simultaneous analysis of multiple samples or conditions (33). As a result, we generated 6 factor-specific networks representing cell-cell interactions among the epithelial and lymphocyte populations. Factors 1, 4, 5, and 6 revealed connections between different subsets of epithelial cells, while factors 2 and 3 highlighted interactions between lymphocytes and epithelial cells (Figure 5C). Focusing on factor 2, which shows signals from epithelial cells to lymphocytes, we observed that interactions between AT2 cells and T cell subsets were stronger than those between AT1 cells and T cells. Additionally, transitional AT2 cells and KRT5^–^KRT17^+^ cells showed strong interactions with both CD4^+^ and CD8^+^/NKT-like cells (Figure 5D). In factor 3 (signals from lymphocytes to epithelial cells), we observed robust communication between CD4^+^ and NK cells and KRT5^–^KRT17^+^ cells (Figure 5D). To determine specific ligand-receptor pairs in these interactions, we explored a bipartite network plot from factors 2 and 3 and identified the chemokine macrophage migration inhibitory factor (*MIF*), which is a ligand for CXCR4 and CD74 on T cells (Figure 5E). These interactions could be important for the recruitment and activation of T cells as well as macrophages (34, 35). Interactions among different epithelial cell populations are depicted in Supplemental Figure 4, A–D, and show a variety of ligand/receptor pairs, including adhesion molecules (*ICAM1*), members of the tissue inhibitor of metalloproteinases (*TIMP*) family, *TGFβ*, and *MMP7*, corroborating an intense tissue remodeling process. Transcripts for MIF were expressed especially by transitional AT2 and KRT5^–^KRT17^+^ epithelial cells (Supplemental Figure 4E). Epithelial cells also expressed varied levels of human leukocyte antigen (*HLA*) class I and II, indicating that these cells could function as antigen-presenting cells to both CD8^+^ and CD4^+^ T cells (Supplemental Figure 4E). Next, we quantified the proportion of *MIF*-positive and *MIF*-negative cells in AT2, transitional AT2, and KRT5^–^KRT17^+^ cells. These 3 populations were classified as *MIF* positive if at least one *MIF* mRNA transcript was detected. We observed that *MIF*-positive cells were abundant across all populations, but the proportion of *MIF*-positive cells was higher in the transitional AT2 and KRT5^–^KRT17^+^ cells (Figure 5F).

An immunofluorescence staining assay for MIF and pan-cytokeratin (Figure 5G) or CD3, CXCR4, and pan-cytokeratin (Figure 5H) showed that MIF was found intracellularly in pan-cytokeratin–positive cells (epithelial cells). Moreover, CXCR4^+^ T cells were found to be abundant in the interstitium of IPF lungs. Together, these results indicate that epithelial cells may reshape the local immune environment by communicating with T cells via the MIF/CXCR4/CD74 axis. Because CXCR4/CD74 and EGFR signaling share several downstream intracellular signals and exhibit crosstalk mechanisms (36), we conclude that these receptors might engage in crosstalk during T cell activation in IPF. We examined expressions of EGFRs, CXCR4, and CD74 in T cell subsets using our CITE-seq analysis, which revealed that the average log expression of ERBB2 was lower compared with CXCR4 and CD74 (Supplemental Figure 5). However, we observed that ERBB2 was detected at high levels in a small group of cells in all populations, but in greater abundance in NKT-like cells. Therefore, the interplay between EGFR, CXCR4, and CD74 signaling pathways may represent a cooperative mechanism driving the activation and functions of several populations of T cells in fibrotic lungs.

## Discussion

In this study, we provide a comprehensive characterization of T cell phenotypes and their spatial distribution within the lungs of IPF patients, shedding light on their potential mechanisms of action during pulmonary fibrosis. Our findings reveal that the predominant localization of T cells within the interstitium may facilitate direct interactions and crosstalk with epithelial cells, contributing to disease progression. We identified 11 populations of T cells in the lungs, including a rare CD56^+^ Treg subset that has the most DEGs in pulmonary fibrosis and exhibits an immunosuppressive-like phenotype. We then showed that CD4^+^ T cells, CD8^+^ GZMB^+^ T cells, and Tregs are located in proximity to epithelial cells in fibrotic lungs, particularly in areas with more severe fibrosis. Furthermore, we highlight the MIF/CXCR4-EGFR signaling axis as an important pathway mediating epithelial–T cell interactions in the fibrotic lung environment. Collectively, these results underscore the relationship between T cell subsets and dysfunctional epithelial cells, such as transitional AT2 cells and KRT5^–^KRT17^+^ cells, and demonstrate how different T cell populations can be activated by environmental signals to carry out their specific functions. Understanding of these subtle interactions among cells could be harnessed to develop therapeutic interventions to ameliorate the chronic injury of epithelial cells in patients with pulmonary fibrosis.

CITE-seq offers a unique opportunity to identify the diverse T cell populations prevailing in the lungs of patients with pulmonary fibrosis. The multimodal analysis of concomitant expression of protein and RNA enables high-resolution characterization of cell phenotypes (12–14), including rare populations observed in our dataset. Our pseudobulk transcriptomic analyses (37–42) rigorously quantify differences in T cell activation between IPF and non-IPF ILDs. In non-IPF ILDs, T cells displayed an activated phenotype dominated by JAK-STAT signaling, a finding that is consistent with well-recognized cytokine-driven immunopathogenic mechanisms of autoimmune diseases (43, 44). In IPF, we observed a distinct activation program regulated by EGFR signaling and TGF-β. EGFR responses in T cells elicit proliferation and activation of diverse effector functions (37–41), including enhancement of FOXP3 expression (45, 46). In our findings, CD56^+^ Tregs were the most activated T cell subset in IPF lungs and were responsive to both EGFR and TGF-β activities, while Tregs were activated mainly by TGF-β. CD56^+^ Tregs have been identified in several TGF-β–enriched environments (47, 48), including hepatocellular carcinoma (47), atherosclerosis (49), and other diseases (48, 50). We observed that the number of CD56^+^ Tregs was increased in the lungs (where EGF signals are more prevalent) but remained low in the peripheral blood of patients, shedding light on a possible tissue-driven origin of these cells. It remains unclear whether CD56^+^ cells acquire a suppressive phenotype that complements Treg function, or whether conventional Tregs upregulate CD56 as they transition to a less suppressive state.

In this study, we identified the chemokine MIF produced by epithelial cells as a potential link to the recruitment of CXCR4^+^/CD74^+^ T cells (as previously shown [refs. 51, 52]). Elevated MIF levels have been consistently reported in the lungs of IPF patients (53, 54), and in studies using bleomycin-induced pulmonary fibrosis models, MIF inhibition has beneficial effects on both inflammation (including TGF-β and TNF levels) and tissue injury (55, 56), emphasizing the role of MIF in fibrotic progression. Our scRNA-seq data showed that *MIF* mRNA was expressed in transitional dysregulated subsets of epithelial cells (57, 58), which showed robust cell-to-cell interactions with CD4^+^ and CD8^+^ T cells. Thus, our cell-to-cell interaction analysis, which is consistent with reports that EGFR and CXCR4/CD74 have common intracellular signals and synergistic effects (59–61), indicates that these pathways may culminate in the final activation of T cells in IPF lungs. Importantly, epithelial cells appear as central players in this process.

The limitations of our study include the fact that lung tissues were obtained from patients with advanced disease, limiting our ability to determine whether these responses are also present in the early stages of pulmonary fibrosis. Our CITE-seq analysis was performed in different batches to minimize the stress on tissue-isolated cells, which can introduce batch effects in single-cell analysis. To address this, we used subject-level pseudobulk analyses and conservative statistical approaches to mitigate these effects on DEGs. Nonetheless, the larger number of DEGs observed in non-IPF ILD samples may in part reflect some batch-related differences. Additionally, our study lacks the statistical power to segregate patients based on treatments or smoking status, preventing us from determining the impact of antifibrotic or antiinflammatory therapies or smoking on T cell functions. Exploring differences in T cell functions between early and late phases of the disease could provide valuable insights into how disease progression influences local T cell responses.

Together, our findings provide important insights into the pathogenesis of IPF and highlight differences and similarities in T cell functions between IPF and other forms of ILD. These studies demonstrate that epithelial cells actively influence lung T cells through conserved communication pathways, and controlling MIF/CXCR4 appears to be a common avenue to block or impair excessive T cell responses. A deeper understanding of how these pathways are increased and progress in fibrosis could lead to therapeutic and translational opportunities aimed at minimizing the risk of epithelial injury in IPF that leads to disease progression.

## Methods

### Sex as a biological variable.

IPF is more common in older adults and males, while autoimmune-mediated ILD is more prevalent in females. In our study, we included a comparable number of age-matched controls and individuals of both sexes, and similar findings are reported for males and females. 

### Subjects and samples.

Tissue samples were obtained from fibrotic lungs (idiopathic pulmonary fibrosis [IPF], interstitial pneumonia with autoimmune features [IPAF], nonspecific interstitial pneumonia [NSIP], and systemic autoimmune rheumatic disease–associated interstitial lung disease [SARD-ILD]) removed at the time of lung transplant surgery at Vanderbilt University Medical Center or non-fibrotic control lungs (declined donors) obtained at the time of organ procurement (Supplemental Table 1). The fibrotic diagnoses were determined according to the American Thoracic Society/European Respiratory Society consensus criteria, as previously described (23).

### Multiplex immunofluorescence in lung tissue.

Tissue microarray sections were generated from paraffin-embedded lung samples of non-diseased controls (*n* = 6) and IPF patients (*n* = 6). Sections were cut at a thickness of 5 μm, placed on poly-lysine–treated coverslips, and incubated in antigen retrieval buffer (Dako Target Retrieval Solution). An additional section was stained with hematoxylin and eosin. Coverslips were incubated overnight with 14 pre-conjugated barcode antibody cocktails (Akoya Biosciences). Slides were placed on the Keyence BZ-X810 microscope stage insert, connected to the PhenoCycler automated fluidics system (Akoya Biosciences). Up to 3 fluorophore-conjugated reporters were added per cycle for hybridization with the antibodies, and 4′,6-diamidino-2-phenylindole (DAPI) was used to label nuclei. Images were captured before dehybridization, and image files were overlaid to form a large region of interest. QuPath (62) was used to segment and annotate cell types. Cells were identified based on marker expression: pan-cytokeratin (epithelial cells), CD4^+^ (CD4^+^ T cells), FOXP3^+^ (Tregs), CD56^+^FOXP3^+^ (CD56^+^ Tregs), CD8^+^ (CD8^+^ T cells), and CD8^+^GZMB^+^ (CD8^+^ GZMB^+^ T cells).

### Spatial proximity analysis of T cells to epithelial cells and established pulmonary fibrosis histopathologies.

Cell type annotations, spatial coordinates, and representative epithelial and immune annotations were utilized from our prior Xenium spatial transcriptomics dataset, which comprised 45 samples (10 unaffected; 35 pulmonary fibrosis diagnoses, including 16 IPF [ref. 26]). Samples were stratified into 3 severity groups (unaffected, less affected, and more affected) based on diagnosis and percentage of tissue pathology as previously described. For each epithelial cell (alveolar: AT1, AT2, transitional AT2, KRT5^–^KRT17^+^, proliferating AT2; airway: basal, goblet [comparable to Secretory_SCGB3A1^+^MUC5B^+^ in the current dataset], respiratory airway epithelial cell [RASC; comparable to Secretory_SCGB3A2^+^], secretory [comparable to Secretory_ SCGB3A2^+^SCGB1A1^+^], multiciliated, pulmonary neuroendocrine cells [PNEC], proliferating airway), the nearest neighbor T cell of any subtype (CD4^+^, CD8^+^, Treg, NK/NKT, proliferating T cell, proliferating NK/NKT) was identified. These data were summarized as the median distance of the nearest neighbor T cell per sample, per epithelial cell subtype. Samples required more than 10 epithelial cells of a given subtype to be included in the analysis. Cell subtypes with fewer than 3 samples in at least 2 severity groups (i.e., PNEC and proliferating airway) were fully removed. Epithelial cells were then split into alveolar and airway groupings to quantify the cumulative number of T cells within 0–50 μm, calculated in 10 μm bins. Results were averaged across all alveolar or airway cells within a sample to calculate the mean number of T cell neighbors at each distance. For the 18 annotation types across 27 samples (7 unaffected; 20 pulmonary fibrosis diagnoses, including 10 IPF), which were selected as previously described (epithelial: normal alveoli, minimally remodeled alveoli, emphysema, hyperplastic alveolar epithelial cells, remodeled epithelium, advanced remodeling, epithelial detachment, remnant alveoli, small airway, large airway, microscopic honeycombing, goblet cell metaplasia; immune: granuloma, mixed inflammation, tertiary lymphoid structure; fibrotic: fibrotic focus, fibrosis, severe fibrosis), we calculated the composition of T cell subtypes inside and within 50 μm of the cells assigned to each annotation instance. For each annotation type, there were 4–81 instances across 2–25 samples and 2–20 donors (mean instances: 28.2; mean samples: 10.8; mean donors: 9.4). Of the annotation types, “normal alveoli,” “minimally remodeled alveoli,” “emphysema,” and “small airway” were characterized in both unaffected and disease, and all other annotations were exclusive to disease. To avoid double counting in cases in which multiple annotation instances shared a 50 μm ring zone, each T cell was only counted once per annotation type.

### Isolation of single-cell suspensions from lungs.

Lung tissue biopsies were washed in PBS, minced, and frozen in RPMI medium containing 10% DMSO until analysis. Minced lungs were thawed at 37°C for 3 minutes, digested in an enzymatic cocktail (collagenase XI and DNase I, type IV [MilliporeSigma] [ref. 63], for CITE-seq, or collagenase I/dispase II for scRNA-seq) (23) using a gentleMACS Octo Dissociator (Miltenyi Inc.) at 37°C, and filtered using 100 μm sterile filters (Miltenyi Biotech).

### Cytometry by time of flight.

Peripheral blood (*n* = 25 IPF [average age 68 ± 7.4]; *n* = 20 controls [average age 66 ± 9.3]) and lung-isolated cells (*n* = 12 IPF and *n* = 2 other ILD [average age 48 ± 7.6]; *n* = 15 controls [average age 33 ± 17]) were processed for cytometry by time of flight (CyTOF) as previously described (11). Raw data were normalized using CyTOF software and further analyzed with FlowJo version 10.10.0 (BD Life Sciences). Live cells were identified by exclusion of cisplatin-positive cells, and viSNE maps were generated using selected CD45^+^CD3^+^ live cells. Subsequently, CD4^+^CD25^+^ cells were gated, and the percentages of CD56^+^ and CD56^–^ cells (in peripheral blood) or CD38^+^ and CD38^–^ cells (in lungs) were determined for each sample.

### Cellular indexing of transcriptomes and epitopes by sequencing (CITE-seq).

Single-cell RNA sequencing/CITE-seq was performed using single-cell suspensions derived from cryopreserved minced lung tissue of control and fibrotic samples. Whole-lung cell suspensions (up to 1 million cells) were incubated with an Fc receptor blocking reagent (TrueStain FcX, BioLegend) for 15 minutes, followed by staining with a panel of flow cytometry antibodies (anti-CD45 [PERCy5.5, 2D1], anti-CD3 [PE-Cy5, HIT3a], and 14 oligo-conjugated TotalSeq-C human antibodies [BioLegend, Supplemental Table 2]) for 30 minutes. Cells were washed 3 times with PBS containing 1% BSA and stained with a viability dye (Zombie Aqua kit, BioLegend). Up to 25,000 viable CD45^+^CD3^+^ cells were sorted per sample using a BD FACSAria and submitted for sequencing on the 10x Genomics Chromium platform. Briefly, 10,000 cells were loaded onto a 10x Chromium single-cell encapsulation chip, and libraries were prepared according to the manufacturer’s instructions. Sequencing was performed on Illumina platforms at a depth of at least 80 million reads per library.

### Analysis of sequencing data.

After alignment, demultiplexing (Cell Ranger 6.0.0/6.1.1), and quality control filtering, we removed genes expressed in fewer than 3 cells, as well as cells with greater than 10% mitochondrial mRNA mapped unique molecular identifiers (UMIs), fewer than 200 identified genes, more than 5,000 identified genes, total RNA counts less than 1,500, or total RNA counts greater than 15,000. Multimodal data (gene expression and protein expression) were integrated using a Muon/Scanpy/Harmony/Multi-Omics Factor Analysis (MOFA)–based workflow, which jointly utilized protein and RNA results for manual cell annotation. The MuData object integrated 93,796 cells and featured 24,719 genes and 15 proteins. Detailed quality control metrics are in Supplemental Table 4.

Whole-lung scRNA-seq from the same subjects who underwent T cell profiling (previously reported in Gene Expression Omnibus GSE135893 and/or GSE227136) was reanalyzed using a similar Scanpy-based workflow, including label transfer from the parent object (GSE227136) using CellTypist (64). Distinct populations of immune (myeloid and lymphoid) and non-immune cells (epithelial, endothelial, and fibroblasts) were identified in the whole-lung RNA-seq dataset. Cell-cell communication between lymphocytes (CD4^+^, CD8^+^/NKT-like, and NK cells) and epithelial cells (alveolar type 1 [AT1], AT2, transitional AT2, KRT5^–^KRT17^+^, and secretory cells) was assessed using LIANA and Tensor-cell2cell (33). In brief, ligand-receptor interaction scores were calculated for each sample using LIANA. These scores were then aggregated into a tensor via the cell2cell framework, with sample and condition incorporated into the tensor dimensions. Tensor decomposition was performed to identify latent communication factors, revealing key sender and receiver cell populations, ligand-receptor pairs, and pathways associated with fibrosis. In the CITE-seq dataset, 11 major T cell clusters were identified. DESeq2 (42) pseudobulk analysis was performed to identify DEGs among T cell populations in IPF compared with control samples. Gene ontology analysis of DEGs was performed using the Reactome pathway database (28).

### Statistics.

Statistical analyses were performed in Python of GraphPad Prism software v8. For comparisons between 2 independent groups, we used a 2-tailed Student’s *t* test for approximately normally distributed data or a 2-tailed Mann-Whitney *U* test for non-normally distributed data. For comparisons among 3 groups, we used 1-way ANOVA (parametric) or the Kruskal-Wallis test (non-parametric). Unless otherwise stated, *P* < 0.05 was considered statistically significant.

### Study approval.

These studies were approved by the Institutional Review Board at Vanderbilt University (IRB 171657, 162138, 060165, 190768).

### Data availability.

Supporting CITE-sequencing data of this study are in the NCBI’s Gene Expression Omnibus database (GEO GSE326573), and all numeric data shown in the graphics are included in the Supporting Data Values file. The codes used for analysis in this paper are available at https://github.com/KropskiLab/tcell_multiomics, commit ID: c04352ca9b121090f80b3aad94e6688b6dc905f4 and cfa84138e4ed1b63b95fbcb04dcfb1ae2f0b0502.

## Author contributions

APMS, JAK, and TSB designed research studies. APMS, JMRB, BDP, and LDS conducted experiments. CJT, TS, CLC, EMW, MB, CMS, LBW, and MLS organized or performed sample collection. APMS, LVK, PIGE, JAK, and NEB performed analysis and interpretation of data. APMS, JAK, AJD, AV, and CJT performed the bioinformatics analyses. APMS, JAK, and TSB wrote the manuscript.

## Conflict of interest

TSB received funding from Boehringer Ingelheim and Bristol Myers Squibb. MLS received funding from Boehringer Ingelheim Pharmaceuticals and personal fees for consulting from Boehringer Ingelheim Pharmaceuticals, Roche, Trevi Therapeutics, PureTech Health, and Insilico Medicine. JAK received funding from Boehringer Ingelheim. LBW received consulting fees from Healios and Novartis and research funding from Bluejay Diagnostics and holds stock in Virtuoso Surgical.

## Funding support

This work is the result of NIH funding, in whole or in part, and is subject to the NIH Public Access Policy. Through acceptance of this federal funding, the NIH has been given the right to make the work publicly available in PubMed Central.

- American Lung Association 1456724 (to APMS).
- NIH 8K12AR084232-24 (to APMS); P01HL172729 (to TSB, MLS, NEB, and JAK); K23HL141539 (to MLS); K08AR080808 and KL2TR002245 (to EMW); and HL158906 (to LBW).

## Supplementary Material

Supplemental data

Supporting data values

## Version 1 09/08/2026

Electronic publication
