# Supplementary material for: Multiomic analysis identifies T cell subsets and mechanisms of epithelial interaction in idiopathic pulmonary fibrosis
Source: JCI Insight. 2026 Sep 8;11(17):e203080. doi: 10.1172/jci.insight.203080 (PMC13564086; doi:10.1172/jci.insight.203080)
Supplement: Supplementary file 1 [file jciinsight-11-203080-s129.pdf]

Table 1: Characteristics of participants

Characteristics	Healthy Controls (n=10)	IPF (n=9)	SARD-ILD (n=5)	IPAF (n=3)	NSIP (n=2)
**Age (years), mean (SD)**	50.3 ±11.9	62.2 ± 5	58.6 ±7	48±7	59.5±5
**Gender, N (%)**					
**Male**	50%	55.5%	20%	33%	50%
**Female**	50%	44.5%	80%	67%	50%
**Smoking history, N (%)**	30%	66%	20%	33%	0%
**Ethnicity, N (%)**					
**European**	80%	77%	80%	67%	100%
**African**	20%	0%	20%	0%	0%
**Latino**	0%	11%	0%	0%	0%
**Other**	0%	0%	0%	33%	0%

IPF= Idiopathic Pulmonary Fibrosis
SARD-ILD = Systemic Autoimmune Rheumatic Disease-associated Interstitial Lung Disease
IPAF= Interstitial pneumonia with autoimmune features
NSIP=nonspecific interstitial pneumonia
Smoking status includes both current and former smokers

Table 2: List of CITE-seq and flow sorting antibodies

Antibody & Clone	Conjugation	Catalog
CD3_totalSeq-C (UCHT1)	CTCATTGTAACTCCT	C0034
CD8_totalSeq-C (SK1)	GCGCAACTTGATGAT	C0046
CD56_totalSeq-C (5.1H11)	TCCTTTCCTGATAGG	C0047
CD4_totalSeq-C (RPA-T4)	TGTTCCCGCTCAACT	C0072
CD25_totalSeq-C (BC96)	TTTGTCCTGTACGCC	C0085
CD45RO_totalSeq-C (UCHL1)	CTCCGAATCATGTTG	C0087
PD1_totalSeq-C (EH12.2H7)	ACAGCGCCGTATTTA	C0088
CD103_totalSeq-C (Ber-ACT8)	GACCTCATTGTGAAT	C0145
CCR7_totalSeq-C (G043H7)	AGTTCAGTCAACCGA	C0148
CTLA4_totalSeq-C (BNI3)	ATGGTTCACGTAATC	C0151
LAG3_totalSeq-C (11C3C65)	CATTTGTCTGCCGGT	C0152
HLADR_totalSeq-C (L243)	AATAGCGAGCAAGTA	C0159
CD57_totalSeq-C (QA17A04)	AACTCCCTATGGAGG	C0168
CD28_totalSeq-C (CD28.2)	TGAGAACGACCCTAA	C0386
KLRG1_totalSeq-C (2F1/KLRG1)	GTAGTAGGCTAGACC	C0250
CD45 (2D1)	PercpCy5.5	368504
CD3 (HIT3a)	Pecy5	300310

Table 3: List of CODEX and immunofluorescent antibodies

Antibody & Clone	Conjugation	Catalog
CD20-BX007 (L26; AKYP0049)	Alexa Fluor 750-RX007	4450018
CD56-BX028 (CAL53; AKYP0118)	Atto 550-RX028	4250087
CD56-BX028 (CAL53; AKYP0118)	Alexa Fluor™ 750-RX028	6450010
FOXP3-BX031 (236A/E7; AKYP0102)	Alexa Fluor 647-RX021	4550071
Granzyme B-BX041 (D6E9W; AKYP0086)	Atto 550-RX041	4250055
CD4-BX003 (EPR6855; AKYP0048)	Alexa Fluor 647-RX003	4550112
CD31-BX001 (EP3095; AKYP0047)	Alexa Fluor 750-RX001	4450017
CD163-BX069 (EPR19518; AKYP0114)	Atto 550-RX069	4250079
CD68-BX015 (KP1; AKYP0050)	Alexa Fluor 647-RX015	4550113
CD45RO-BX017 (UCHL1; AKYP0059)	Atto 550-RX017	4250023
HLA-DR-BX033 (EPR3692; AKYP0063)	Alexa Fluor 647-RX033	4550118
Pan-Cytokeratin-BX019 (AE-1/AE-3; AKYP0053)	Alexa Fluor 750-RX019	4450020
CD8-BX026 (C8/144B; AKYP0028)	Atto 550-RX026	4250012
CD45-BX021 (D9M8I; AKYP0074)	Alexa Fluor 647-RX021	4550121
CD3 (SP162)	Primary antibody	ab135372
CXCR4 (Polyclonal)	Primary antibody	PA1-21626
Pan-Cytokeratin (Polyclonal or AE1/AE3l)	Primary antibody	AB9377 or CF190321
MIF (2Ar3)	Primary antibody	MA1-20881

Table 4: QC metrics and number of cells per subject

Subject	Number of Cells	Mean Genes/ Cell	Mean UMIs/ Cell	CD4 T_{CM}	CD4 T_{EM}	CD4 T_{RM}	CD4 T_{REG}	CD56 T_{REG}	CD8 T_{RM}	CD8 T_{EM} GZMK	NKT	γδ T	Cytot CD4	Cytot CD8	Tobacco use
Control 1	1967	1283.62	2268.72	384	567	81	71	10	130	153	151	151	102	77	No
Control 2	1086	1381.18	2352.48	216	306	139	41	5	111	73	26	58	42	44	No
Control 3	261	1083.37	1989.29	42	45	7	22	2	8	8	3	34	20	30	No
Control 4	631	1261.4	2168.24	60	101	21	48	5	36	44	17	64	38	36	No
Control 5	3042	1267.62	2033.2	179	197	70	30	11	309	258	364	885	245	421	Yes
Control 6	3647	1245.93	2300.71	367	281	62	24	29	251	277	569	531	851	354	No
Control 7	2439	1302.3	2624.4	451	431	102	109	7	284	298	166	145	125	87	Yes
Control 8	2757	1247.08	2420.22	236	352	82	34	17	259	199	413	455	212	447	Yes
Control 9	4666	1465	3288.85	188	188	88	42	37	483	540	1165	512	211	1169	No
Control 10	5109	1474.52	3391.36	664	738	389	79	76	310	872	806	373	273	482	No
IPF 1	2966	1387.93	3260.1	633	293	81	242	31	610	430	74	227	68	238	No
IPF 2	848	1286.9	2324.15	89	74	23	92	14	233	34	133	30	14	15	Yes
IPF 3	1735	1306.07	2229.6	156	138	28	221	10	116	135	19	403	127	283	Unknown
IPF 4	872	1263.41	2341	117	110	29	177	13	48	39	11	104	56	52	Yes
IPF 5	4196	1382.06	2879.38	352	714	220	206	29	477	346	135	377	728	496	No
IPF 6	2108	1252.9	2676.36	273	197	39	139	18	176	172	171	195	307	268	Yes
IPF 7	2173	1342.17	2883.16	655	419	72	181	14	354	133	18	38	62	40	Yes
IPF 8	3351	1359.66	3187.67	359	490	208	138	18	536	185	483	304	186	394	Yes
IPF 9	1023	1441.9	3439.27	334	134	70	185	19	176	41	22	1	16	6	Yes
Non-IPF ILD 1	4631	1694.7	3300	390	256	76	100	14	702	512	152	901	859	567	Yes
Non-IPF ILD 2	5279	1224.66	3079.25	339	272	57	37	67	188	528	1406	482	968	898	No
Non-IPF ILD 3	5957	1443.06	2859.51	629	560	124	102	20	974	604	253	768	1400	438	No
Non-IPF ILD 4	3243	1082.68	2586.44	320	206	212	120	24	1164	189	691	71	67	144	No
Non-IPF ILD 5	4283	1302.93	3330.96	684	523	437	167	23	1146	242	436	65	227	299	No
Non-IPF ILD 6	4559	1292.93	3130.52	1137	446	177	349	87	288	477	382	438	327	390	No
Non-IPF ILD 7	9780	1492.22	3605.41	581	623	135	210	38	598	1036	661	1842	1182	2470	No
Non-IPF ILD 8	3841	1432.72	3620.3	522	332	149	430	31	898	326	367	231	111	347	Yes
Non-IPF ILD 9	6633	1592.66	3773.09	718	394	192	621	163	614	523	496	983	569	1182	No
Non-IPF ILD 10	698	1328.74	3164.86	28	15	6	30	12	32	98	71	96	100	198	No

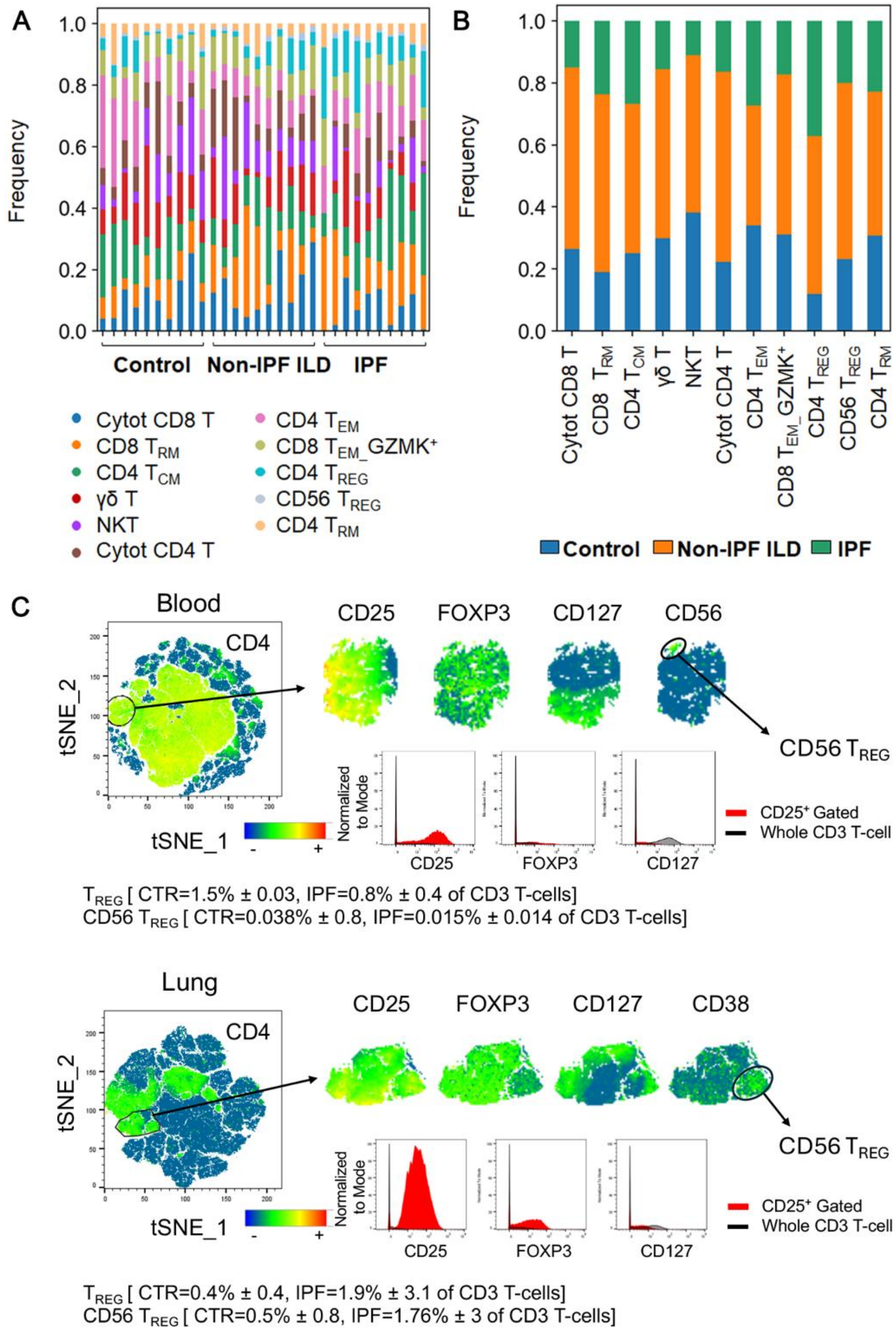

Supplemental Figure 1: T-cell type frequency in the lungs and identification of T_{REGs} and CD56 T_{REGs} using CyTOF. A-B. Bar charts illustrate the frequency of the indicated T-cell subsets determined by CITE-seq analysis of CD3-isolated T-cells from controls (CTR), non-IPF ILD, and IPF lungs. Data are grouped by individual subjects (**A)** or by the three donor groups (**B**) [controls, non-IPF ILD, and IPF]. **C.** viSNE map shows the distribution of $CD4^+CD25^+$ T-cells and the expression of specific markers (including FOXP3, CD127, CD56, or CD38), based on cytometry by time of flight (CyTOF) analysis. Cells were isolated from either the peripheral blood (n=45) or the lungs (n=39) of controls and IPF patients. The percentages of $CD4^+CD25^+$ and $CD4^+CD25^+CD56^+$ or $CD4^+CD25^+CD38^+$ cells are shown relative to the total $CD3^+$ T-cell population for each group.

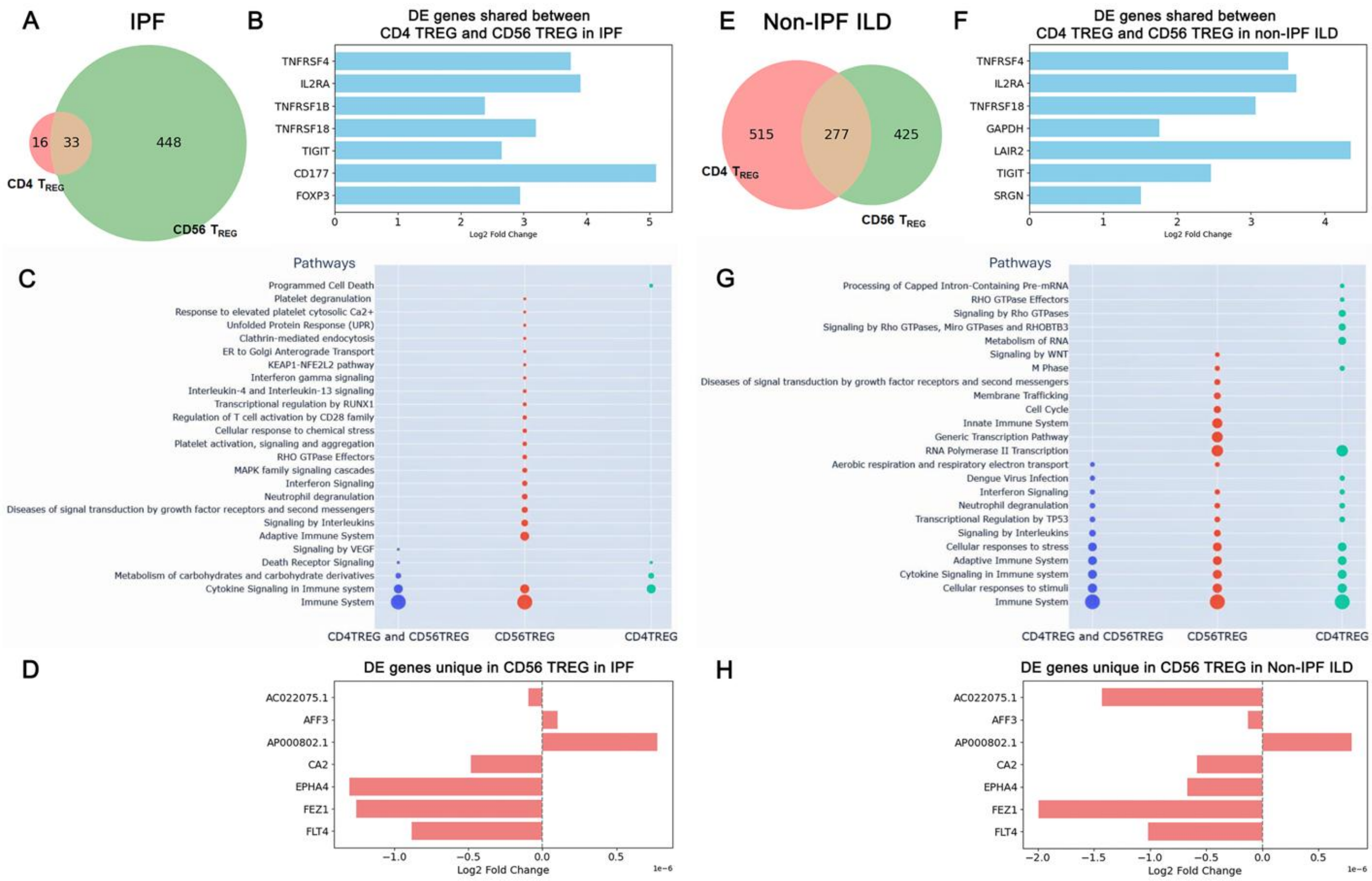

Supplemental Figure 2: Characterization of CD4 T_{REGs} and CD56 T_{REGs} in IPF. A. Venn diagram indicating the numbers of differentially expressed genes in IPF CD4 T_{REGs} and CD56 T_{REGs} in IPF compared to control samples. DEGs were identified using PyDEseq2 pseudobulk analysis, with thresholds of adjusted p-value (padj < 0.05) and log2 fold change > ±1. **B.** Bar chart showing log fold change of the most significant DEGs shared between CD4 T_{REGs} and CD56 T_{REGs} in IPF samples. **C.** Pathway analysis from shared and uniquely expressed DEGs in CD4 T_{REGs} and CD56 T_{REGs} from IPF samples was performed using Reactome. **D.** Bar chart showing the log fold change of the most significant DEGs only expressed by CD56 T_{REGs} in IPF. **E.** Venn diagram indicating the numbers of differentially expressed genes in CD4 T_{REGs} and CD56 T_{REGs} in non-IPF ILD compared to control samples. DEGs were identified using PyDEseq2 pseudobulk analysis, with thresholds of adjusted p-value (padj < 0.05) and log2 fold change > ±1. **F.** Bar chart showing log fold change of the most significant DEGs shared between CD4 T_{REGs} and CD56 T_{REGs} in non-IPF ILD samples. **G.** Pathway analysis from shared and uniquely expressed DEGs in CD4 T_{REGs} and CD56 T_{REGs} from non-IPF ILD samples was performed using Reactome. **H.** Bar chart showing log fold change of the most significant DEGs only expressed by CD56 T_{REGs} in non-IPF ILD

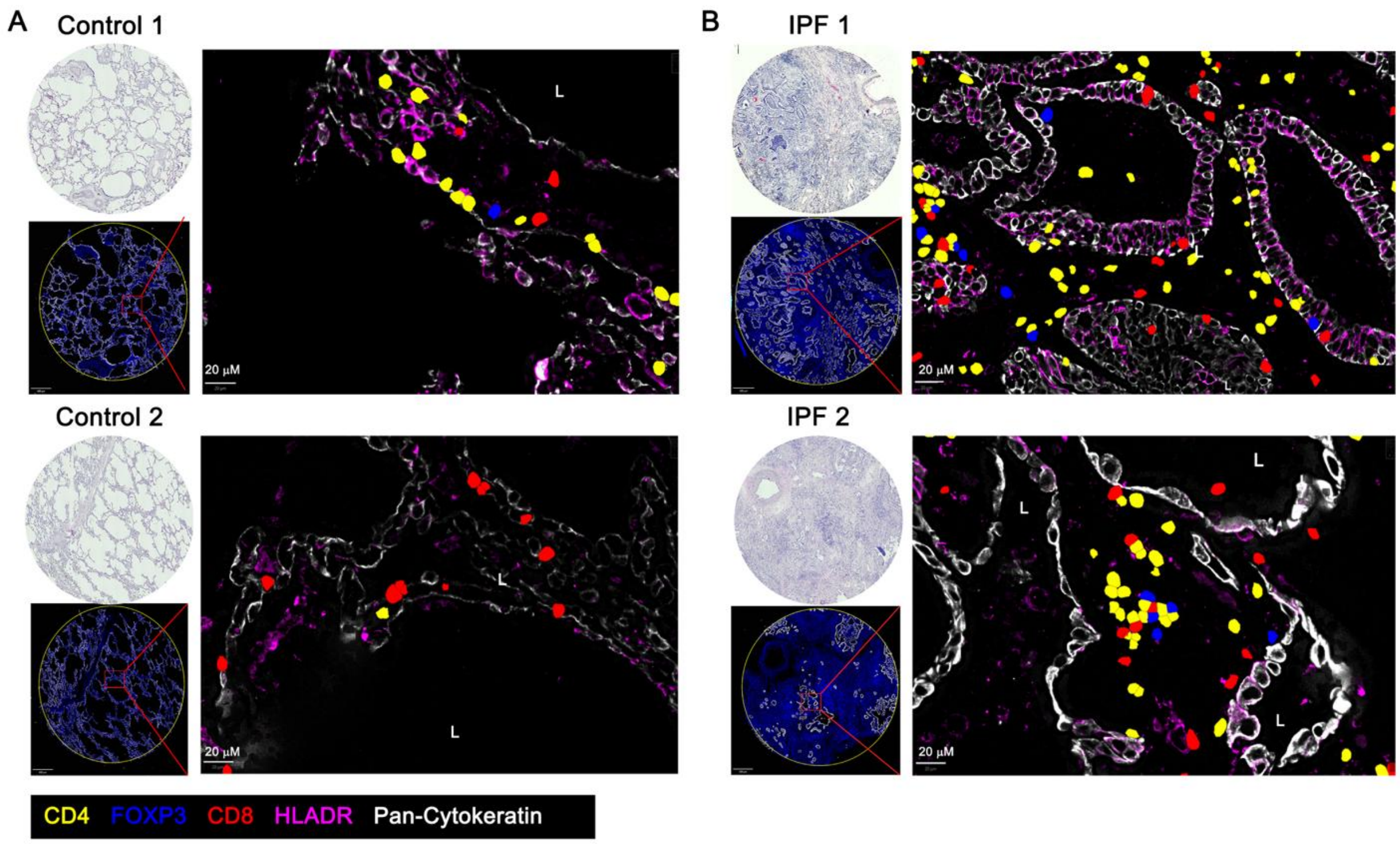

Supplemental Figure 3. CODEX spatial imaging of control and IPF lung. A–B. Multiplexed CODEX images from lung sections of two representative control donors and two representative IPF donors (n=6 for each group). For each section, the H&E image is shown (upper left) alongside the corresponding DAPI (nucleus) and pan-cytokeratin expression (lower left). A representative region of interest highlights epithelial cells (pan-cytokeratin, white), CD4 T cells (CD4, yellow), regulatory T cells (FOXP3, blue), and CD8 T cells (CD8, red), and HLA-DR (pink), illustrating the abundance of T-cells in the interstitium and the abundance of HLA-DR expression by epithelial cells. (L denotes the alveolar/airway lumen). Scale bar: [20 µm].

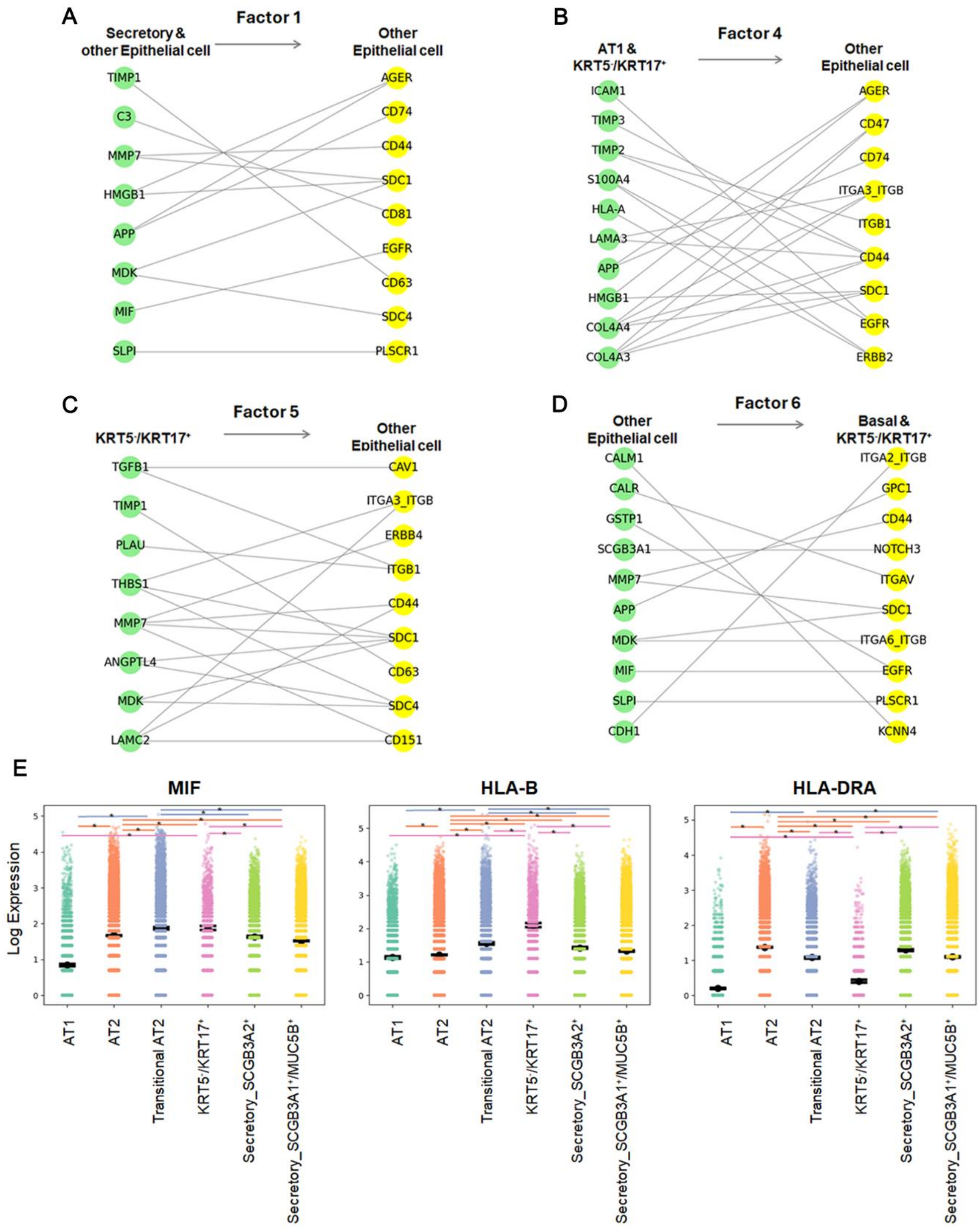

Supplemental Figure 4: Epithelial ligand-receptor interactions and expression of MIF and HLA by the different epithelial populations. A-D. Bipartite network plots illustrating significant ligand-receptor interactions associated with Factors 1 (**A**), 4 (**B**), 5 (**C**), and 6 (**D**), representing epithelial-to-epithelial communication. In the network plots, nodes correspond to ligands and receptors, while edges represent interactions with weights greater than 0.005. **E.** A strip and point plot depicting the log-fold expression of *MIF*, *HLA-B*, and *HLA-DRA* across the indicated

epithelial cell subsets. Statistical significance was determined using the Kruskal-Wallis test ($p < 0.05$), followed by pairwise comparisons using Tukey's test for pairwise comparisons.

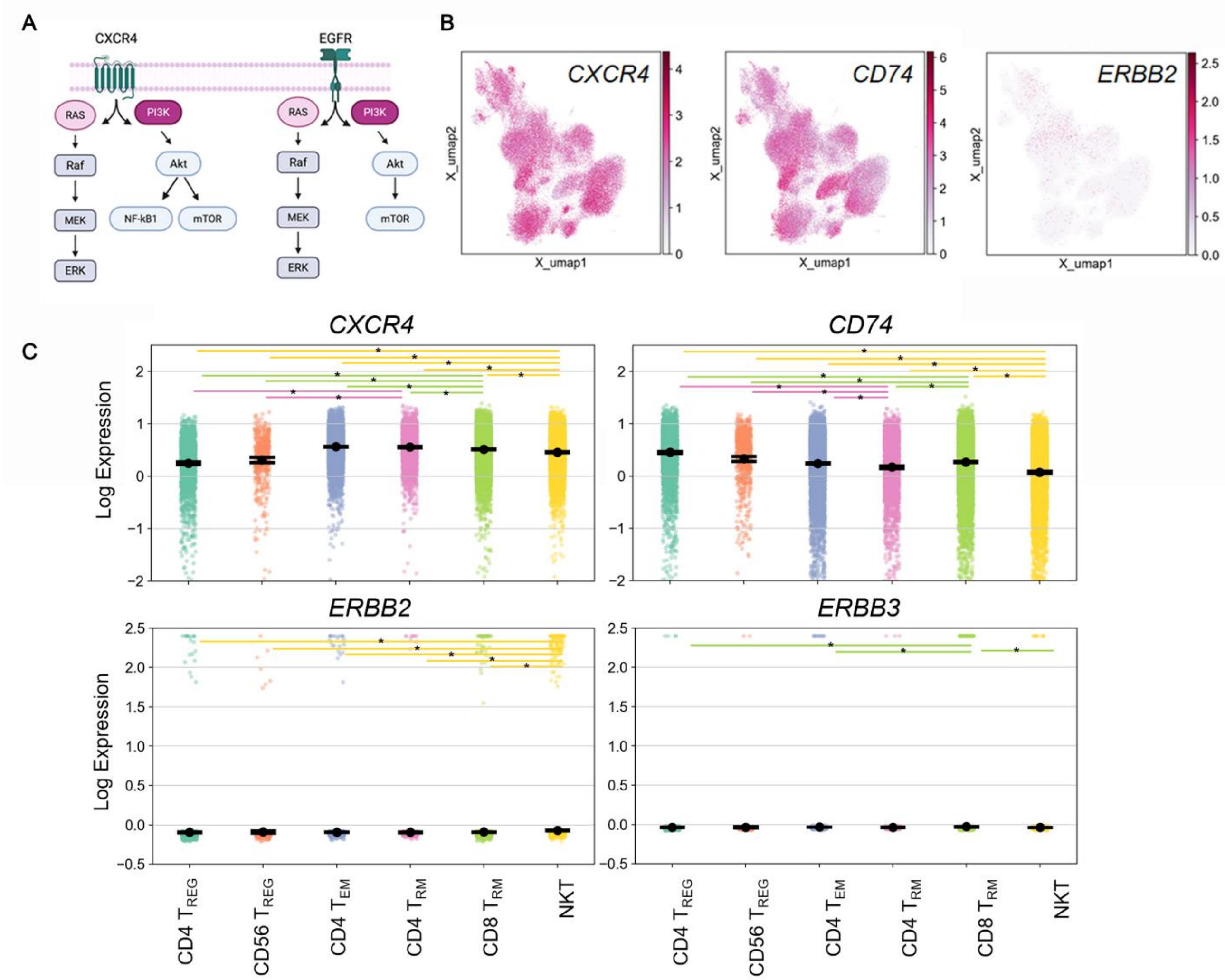

Supplemental Figure 5: CXCR4/CD74 expression pattern in lung T-cell subsets. A. Schematic workflow illustrating the intracellular signaling following CXCR4 or EGFR binding. **B.** UMAP plots depicting the expression of *CXCR4*, *CD74*, and ERBB across T-cell subsets. **C.** Strip and point plot showing the log-fold expression of *CXCR4*, *CD74*, *ERBB2*, and *ERBB3* across the indicated T-cell types. Statistical significance was determined using the Kruskal-Wallis test ($p < 0.05$), followed by pairwise comparisons using Tukey's test for pairwise comparisons.
